# Distributed Node Scheduling with Adjustable Weight Factor for Ad-hoc Networks

**DOI:** 10.3390/s20185093

**Published:** 2020-09-07

**Authors:** Wonseok Lee, Taehong Kim, Taejoon Kim

**Affiliations:** School of Information and Communication Engineering, Chungbuk National University, Chungju 28644, Korea; wonseoklee@cbnu.ac.kr (W.L.); taehongkim@cbnu.ac.kr (T.K.)

**Keywords:** distributed scheduling, weight factor, ad-hoc network, fairness

## Abstract

In this paper, a novel distributed scheduling scheme for an ad-hoc network is proposed. Specifically, the throughput and the delay of packets with different importance are flexibly adjusted by quantifying the importance as weight factors. In this scheme, each node is equipped with two queues, one for packets with high importance and the other for packets with low importance. The proposed scheduling scheme consists of two procedures: intra-node slot reallocation and inter-node reallocation. In the intra-node slot reallocation, self-fairness is adopted as a key metric, which is a composite of the quantified weight factors and traffic loads. This intra-node slot reallocation improves the throughput and the delay performance. Subsequently, through an inter-node reallocation algorithm adopted from LocalVoting (slot exchange among queues having the same importance), the fairness of traffics with the same importance is enhanced. Thorough simulations were conducted under various traffic load and weight factor settings. The simulation results show that the proposed algorithm can adjust packet delivery performance according to a predefined weight factor. Moreover, compared with conventional algorithms, the proposed algorithm achieves better performance in throughput and delay. The low average delay while attaining the high throughput ensures the excellent performance of the proposed algorithm.

## 1. Introduction

An ad-hoc network, as a group of wireless mobile nodes, can be implemented in various forms, including wireless mesh networks, wireless sensor networks, mobile ad-hoc networks, and vehicle ad-hoc networks [1,2]. Ad-hoc networks can provide flexible communication even when it is not possible to install new infrastructure or use existing infrastructure due to geographical and cost restrictions [3]. Ad-hoc networks have the advantage of node communication with other nodes without a base station. Moreover, they also have the features of self-forming and self-healing. Accordingly, they are adopted in various applications, such as battlefield situations, where topology changes frequently, disaster relief, environmental monitoring, smart space, medical systems, and robot exploration [4,5,6,7,8].

Unlike mobile communication networks, which allow centralized resource scheduling, an ad-hoc network requires distributed scheduling based on the information exchanged among nodes. A major problem with distributed node scheduling is packet collisions among nodes if resources are not efficiently distributed, which can lead to significant throughput degradation [9]. Considering these characteristics, supporting quality of service (QoS) through distributed scheduling is a very challenging task. QoS support for high- and low-priority data is essential in various applications. For instance, on a battlefield, a commander’s orders must be delivered as soon as possible. In addition, for environmental monitoring, it is necessary to send emergency disaster information, such as an earthquake alert, to a destination node with very high priority [10].

The nodes of an ad-hoc network consume a lot of energy in sensing data and processing high-priority packet. However, in many situations, it is difficult to replace or recharge the battery of the nodes. Accordingly, it is important to increase energy efficiency and to enhance overall network lifetime through clustering, transmission power control, and efficient network information exchange [11,12,13,14,15,16]. Fairness and load balancing among nodes also have a great influence on the battery lifetime and the connectivity of the entire network. However, low fairness among nodes due to inefficient resource allocation causes increased packet collisions and packet retransmission to some nodes, and these detrimental effects reduce the battery lifetime. Meanwhile, some other nodes will be allocated an unnecessarily much amount of resources, resulting in severe inefficiency for the entire network. Hence, resource allocation for an ad-hoc network is a very important and challenging issue.

Fairness measurements can be categorized into qualitative and quantitative methods, depending on whether the fairness can be quantified. Qualitative methods cannot quantify fairness to an actual value, but they can judge whether a resource allocation algorithm achieves a fair allocation. Maximum-minimum fairness [17,18] and proportional fairness [19] are qualitative methods. Maximum-minimum fairness aims to achieve a max-min state, where the resources allocated to a node can no longer be increased without reducing the resources allocated to neighboring nodes. Proportional fair scheduling maximizes the log utility of the whole network by preferentially scheduling nodes with the highest ratios of currently achievable rates to long-term throughput. Measuring the fairness of an entire network is also an important issue. Jain’s fairness index [20] is a quantitative fairness measurement method, however, it cannot measure the fairness of nodes to which a weight factor is assigned.

In this paper, a distributed scheduling algorithm, which takes weight factors and traffic load into account, is proposed. In the proposed algorithm, self-fairness [21] is adopted for resource reallocation. Increment of self-fairness means that resources are fairly allocated to nodes proportionally to the weight of each node. Therefore, even in the distributed scheduling which supports packets with different importance, if the slot allocation for each node is adjusted to the direction of increasing self-fairness, the overall performance of the network can be significantly increased. Moreover, the proposed algorithm adjusts throughput and delay based on the assigned weight factor rather than an absolute distinction between high-priority packets and low-priority packets.

The contribution of this work is summarized as follows:

- A novel distributed scheduling scheme for an ad-hoc network is proposed, where both the load-balancing among neighboring nodes and the preferential processing for high importance packets are considered.
- An intra-node slot reallocation algorithm is proposed. Each node is equipped with multiple queues, and this algorithm re-arranges the slot allocation between the queues inside a node. Moreover, this algorithm enables a flexible adjustment of throughput and delay, reflecting assigned weight factors.
- Self-fairness for packets with unequal importance is introduced. This metric incorporates both the weight factor and traffic load. The metric plays an important role in achieving a fairness among the packets with the same weight factor and in supporting service differentiation among packets with different weight factors. It is validated that the proposed scheduling scheme substantially increases the performance of the network.
- It is confirmed that the proposed node scheduling outperforms the absolute priority-based scheduling scheme in terms of delay and throughput. This result is supported by thorough simulation studies accommodating various operation scenarios.

The remainder of this paper is organized as follows: Section 2 describes the various distributed resource allocation medium access control (MAC) protocols proposed in the literature. Section 3 describes the proposed algorithm. In Section 4, the performance of the proposed algorithm is analyzed based on an extensive simulation study, and, finally, Section 5 presents some observational conclusions.

## 2. Related Works

In [22], the authors proposed a distributed randomized (DRAND) time division multiple access (TDMA) scheduling algorithm, which is a distributed version of the randomized (RAND) time slot scheduling algorithm [23]. DRAND operates in a round-by-round manner and it does not require time synchronizations on the round boundaries, resulting in energy consumption reduction. In this scheme, there are four states for each node: IDLE, REQUEST, GRANT, and RELEASE. Each node is assigned a slot that does not cause a collision within the 2-hop neighboring nodes by sending a state message to the neighboring nodes. The basic idea of the deterministic distributed TDMA (DD-TDMA) [24] is that each node collects information from its neighboring nodes to determine slot allocations. DD-TDMA is superior to DRAND in terms of running time and message complexity. This feature increases energy efficiency because DD-TDMA does not need to wait for a GRANT message, which is transmitted as a response of REQUEST message and it contains a slot allocation permission for unused slots. However, DRAND and DD-TDMA do not consider load balancing and fairness among the nodes.

Algorithms for allocating resources based on the states of networks and nodes were proposed in [25,26,27,28]. In [25], a load balancing algorithm for TDMA-based node scheduling was proposed. This scheme makes the traffic load semi-equal and improves fairness in terms of delay. In adaptive topology and load-aware scheduling (ATLAS) [26], nodes determine the amount of resources to be allocated through resource allocation (REACT) algorithms, where each node auctions and bids on time slots. Each node acts as both an auctioneer and a bidder at the same time. During each auction, an auctioneer updates an offer (maximum available capacity) and a bidder updates a claim (capacity to bid in an auction). Through this procedure, resources are allocated to the nodes in a maximum-minimum manner [17]. In [27], an algorithm consisting of two sub-algorithms was proposed. The first is a fair flow vector scheduling algorithm (FFVSA) aiming to improve fairness and optimize slot allocation by considering the active flow requirements of a network. FFVSA uses a greedy collision vector method that has less complexity than the genetic algorithm. The second is a load balanced fair flow vector scheduling algorithm (LB-FFVSA), which increases the fairness of the amount of allocated resources among nodes. In [28], the fairness among nodes was improved in terms of energy consumption through an upgraded version of DRAND. Energy-Topology (E-T) factor was adopted as a criterion for allocating time slots, and E-T-DRAND algorithm was proposed to request time slots. Instead of the randomized approach of DRAND, E-T-DRAND algorithm provides high priority to the nodes with high energy consumption and low residual energy due to the large number of neighboring nodes. E-T-DRAND balances the energy consumption among nodes and enhances scheduling efficiency. Each node determines the number of slots to be reallocated using the number of packets accumulated in the queue of its 1-hop neighboring nodes and the number of allocated slots for these nodes. The slot reallocation procedure must check whether a slot is shared by nodes within 2-hop distance. As a result, the load between nodes becomes semi-equal, and the nodal delay is reduced.

In [29,30,31,32,33], scheduling schemes considering priority were proposed. In [29], for the purpose of reducing delay of emergency data, energy and load balanced priority queue algorithm (ELBPQA) was proposed. In this scheme, four different priority levels are defined according to the position of a node in a network. In [30], the highest priority is given to real-time traffic, and the other priority levels are given to non-real time traffics. In order to reduce the end-to-end delay, the packets with the highest priority are processed in a preemptive manner. In [31], priority- and activity-based QoS MAC (PAQMAC) was proposed. In this scheme, the active time of traffic is dynamically allocated according to priority. Specifically, by adopting a distributed channel access scheme, the packet with high priority have reduced back-off and wait times. In [32], I-MAC protocol, which combines carrier sense multiple access (CSMA) and TDMA schemes, was proposed to increase the slot allocation for nodes with high priority. I-MAC consists of a set-up phase and a transmission phase. The set-up phase consists of neighbor discovery, TDMA time-slot allocation using a distributed neighborhood information-based (DNIB) algorithm, local framing for reuse of time slots, and global synchronization for transmission. Nodes with high priority reduce back-off time to increase the opportunity of winning slot allocation, and nodes with the same priority compete for slot allocation. This scheme reduces the energy consumption of nodes with high priority.

In [33], a QoS-aware media access control (Q-MAC) protocol composed of both intra-node and inter-node scheduling was proposed. Intra-node scheduling determines the priority of packets arriving at the queue of a node. Priority is determined according to the importance of packets and the number of hops to a destination node. Q-MAC consists of five queues, where a queue called an instant queue transmits packets as soon as they arrive. The remaining queues transmit packets following the maximum-minimum fairness principle. Inter-node scheduling is a scheme of data transmission among nodes sharing the same channel. A power conservation MACAW (PC-MACAW) protocol based on the multiple access with collision avoidance protocol for Wireless LANs (MACAW) is applied to schedule data transmission. Q-MAC guarantees QoS through dynamic priority assignment; however, latency can be increased due to heavy computational complexity [34].

A comparative analysis of the protocols mentioned in this section is summarized in Table 1. It is largely classified into with and without prioritizations. In the load-balancing classification, “High” means the clear load-balancing by adopting max-min fairness criterion; “Medium” is an indirect load-balancing method by adjusting idle time and access time; and “Low” is the case where the load-balancing method and its effects are not clearly addressed. In the weight factor classification, “No” is strict priority without quantitative values, and PAQMAC and Q-MAC assign quantitative weight values to packets. 

One of the representative fairness measurement methods is Jain’s fairness index, which is a value range (0, 1), and the closer it is to 1 the fairer it is [20]. Jain’s fairness index can measure the fairness of an entire system in a relatively simple way, but it cannot measure the fairness of nodes to which a weight factor is assigned. In [21], the authors proposed a quantitative fairness measurement method applicable to scheduling algorithms with unequal weight factors.

## 3. Proposed Node Scheduling with Weight Factor

Instead of conventional absolute priority-based scheduling, an adjustable and flexible scheduling scheme is proposed. This scheme reallocates slots by taking the weights assigned to the queues of nodes into account. Specifically, intra-node scheduling, which reallocates slots between the queues for high- and low-importance packets, is introduced. Then, it is followed by inter-node scheduling adopted from [25], which reallocates slots among neighboring nodes to increase the fairness measured in terms of traffic load.

**Figure 1:**
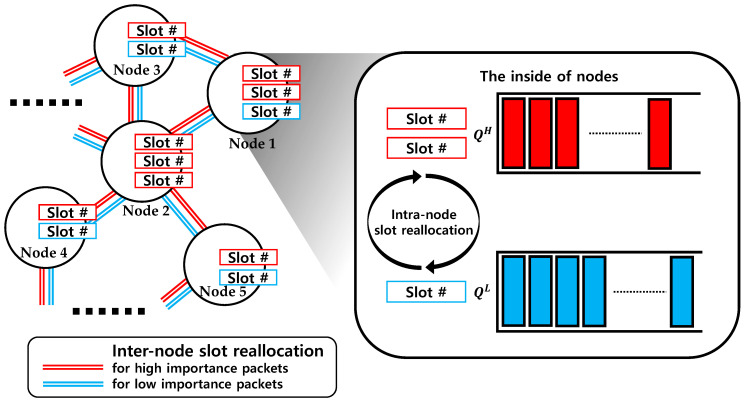
Intra-node slot reallocation and inter-node slot reallocation of the proposed scheduling algorithm.

The proposed algorithm consists of three steps: (1) free time slot allocation, which is a process of allocating the initialized slots (unallocated empty slots) to packets; (2) the intra-node slot reallocation algorithm, which exchanges slots between the queues of a node with different importance values using self-fairness; and (3) the inter-node slot reallocation among 1-hop neighbors using a load balancing algorithm (slot exchange between queues with the same importance). The procedure of this algorithm is depicted in Figure 1.

All the nodes have two types of queues for storing packets of different importance. $Q^{H}$ and $Q^{L}$ are queues for high- and low-importance packets, respectively. $Q^{A},\;A\in \left\{H,\;L\right\}$ represent $Q^{H}$ or $Q^{L}$ according to the indicator A, respectively. In the following, A is used as an indicator representing importance. The number of slots required to transmit all the packets at $Q^{A}$ of node i at frame time t is represented by $q_{t}^{\left(A,i\right)}$, and the number of slots assigned to $Q^{A}$ of node i at frame time t for packet transmission is represented by $p_{t}^{\left(A,i\right)}$. Assuming that the packet and the slot sizes are the same, $q_{t}^{\left(A,i\right)}$ is equal to the number of packets in $Q^{A}$. $p_{t}^{\left(A,i\right)}/q_{t}^{\left(A,i\right)}$ is the inverse load of $Q^{A}$ and expressed as $X_{t}^{\left(A,i\right)}=p_{t}^{\left(A,i\right)}/q_{t}^{\left(A,i\right)}$.

Free time slot allocation requires REQUEST and RELEASE messages exchanges, as in DRAND. The number of packets to be transmitted by node i is $q_{t}^{\left(H,i\right)}$ + $q_{t}^{\left(L,i\right)}$, and node i with $q_{t}^{\left(H,i\right)}+q_{t}^{\left(L,i\right)}>0$ can be allocated slots that are not reserved by the nodes within 2-hop distance. Note that the nodes within 2-hop distance cannot reuse time slot to avoid packet collisions and this reuse can be prevented by slot reallocation between 1-hop nodes. Node i allocates as many as $q_{t}^{\left(H,i\right)}$ slots to $Q^{H}$ and increases $p_{t}^{\left(H,i\right)}$ by the number of allocated slots. If $q_{t}^{\left(H,i\right)}=p_{t}^{\left(H,i\right)}$, $Q^{H}$ does not need to be allocated more slots; accordingly, the slots are allocated to $Q^{L}$, and $p_{t}^{\left(L,i\right)}$ is increased. Afterwards, $p_{t}^{\left(H,i\right)}$ and $p_{t}^{\left(L,i\right)}$ are reallocated through the intra-node slot reallocation algorithm. If both $Q^{H}$ and $Q^{L}$ are allocated as many as $q_{t}^{\left(H,i\right)}$ and $q_{t}^{\left(L,i\right)}$, no more slots are assigned.

In the intra-node slot reallocation, a self-fairness index is used to reallocate packets between $Q^{H}$ and $Q^{L}$ of each node. Self-fairness is a measure of how fairly an amount of "resources" is assigned to a particular node by considering the weight assigned to that node. In this measurement, the resource can be bandwidth, time slots, etc. The proposed algorithm uses inverse load $X_{t}^{\left(A,i\right)}$ as a resource for self-fairness measurement. 

In the proposed algorithm, self-fairness applies to two different queues of each node. Hence, each node has two self-fairness values for its two queues ($Q^{H}$ and $Q^{L}$). The self-fairness value for $Q^{A}$ of node i is denoted by $F_{t}^{\left(A,i\right)}$ and defined as it is presented in Equations (1)–(3) [21]:

(1) $$F_{t}^{\left(A,i\right)}=\frac{\log\;\left(\varphi_{t}^{\left(A,i\right)}\right)}{\log\left(r^{\left(A,i\right)}/r_{Tot}^{\left(A,i\right)}\right)},A\in \left\{H,L\right\}$$

(2) $$\varphi_{t}^{\left(A,i\right)}=\frac{X_{t}^{\left(A,i\right)}}{\sum_{k\in N_{i}}X_{t}^{\left(H,k\right)}+\sum_{k\in N_{i}^{\left(1\right)}}X_{t}^{\left(L,k\right)}}$$

(3) $$r_{Tot}^{\left(A,i\right)}=\underset{k\in N_{i}}{\sum }r^{\left(H,k\right)}+r^{\left(L,k\right)}$$

where $\varphi_{t}^{\left(A,i\right)}$ is the ratio of resources allocated to $Q^{A}$ at node i to the sum of resource allocated to $Q^{H}$ and $Q^{L}$ at 1-hop neighboring nodes, $N_{i}$ is a set of 1-hop neighbor nodes of node i, $r^{\left(A,i\right)}$ is the weight assigned to $Q^{A}$ of node i, and $r_{Tot}^{\left(A,i\right)}$ is the sum of the weights of 1-hop neighboring nodes. When the weight is high, more slots are allocated to increase the inverse-load, resulting in a fairer resource allocation. By setting $r^{\left(H,i\right)}>r^{\left(L,i\right)}$, more important packets are allocated more slots than less important packets. Accordingly, $F_{t}^{\left(A,i\right)}$ is a quantitative value for $Q^{A}$ of node i, indicating whether the load of $Q^{A}$ is high or low considering the weight assigned. Therefore, it is used as an index to compare the fairness of slot allocation with unequal weight factor.

When $F_{t}^{\left(A,i\right)}=1$, the allocation is in the fairest state. When the amount of slots allocated is small compared to the assigned weight factor, $F_{t}^{\left(A,i\right)}>1$ can be satisfied because $\varphi_{t}^{\left(A,i\right)}\in \left[0,\;1\right]$. In this case, it is necessary to gain more slots from the other queue. In the opposite case, if too many slots are allocated, $F_{t}^{\left(A,i\right)}<1$ can be satisfied, and $Q^{A}$ must release its own slots. When a slot is gained, $p_{t}^{\left(A,i\right)}$ and $\varphi_{t}^{\left(A,i\right)}$ will increase, resulting in a decrement of $F_{t}^{\left(A,i\right)}$. In contrast, when a slot is released, $F_{t}^{\left(A,i\right)}$ increases. The intra-node slots reallocation algorithm adjusts $F_{t}^{\left(H,i\right)}$ and $F_{t}^{\left(L,i\right)}$ to be as close to 1 as possible, which improves the self-fairness. Specifically, when $F_{t}^{\left(H,i\right)}>F_{t}^{\left(L,i\right)}$, the slots allocated to $Q^{L}$ are released to $Q^{H}$, and vice versa when $F_{t}^{\left(H,i\right)}<F_{t}^{\left(L,i\right)}$. The algorithm for $F_{t}^{\left(H,i\right)}>F_{t}^{\left(L,i\right)}$ is detailed in Algorithm 1.

**Algorithm 1.** Increasing $Q^{H}$ slot allocation1: **for** all node *i*
**do**2: **if**
$q_{t}^{\left(H,i\right)}$!=03:  Calculate
$F_{t}^{\left(H,i\right)}$
4: **end if**5: **if**
$q_{t}^{\left(L,i\right)}$!=06:  Calculate
$F_{t}^{\left(L,i\right)}$
7: **end if**8: **if**
$F_{t}^{\left(H,i\right)}>F_{t}^{\left(L,i\right)}$
9:  **if**
$p_{t}^{\left(L,i\right)}>0$10:   ${\hat{F}}_{t}^{\left(H,i\right)}\leftarrow$
$p_{t}^{\left(H,i\right)}+1$11:   ${\hat{F}}_{t}^{\left(L,i\right)}\leftarrow$
$p_{t}^{\left(L,i\right)}-1$12:   ${\hat{𝓕}}_{t}^{i}\leftarrow \sqrt{{\left(1-{\hat{F}}_{t}^{\left(H,i\right)}\right)}^{2}+{\left(1-{\hat{F}}_{t}^{\left(L,i\right)}\right)}^{2}}$
13:   ${𝓕}_{t}^{i}\leftarrow \sqrt{{\left(1-F_{t}^{\left(H,i\right)}\right)}^{2}+{\left(1-F_{t}^{\left(L,i\right)}\right)}^{2}}$
14:  **end if**
15:  **while**
$F_{t}^{i}>{\hat{F}}_{t}^{i}$
**do**
16:   $p_{t}^{\left(H,i\right)}\leftarrow p_{t}^{\left(H,i\right)}+1$
17:   $p_{t}^{\left(L,i\right)}\leftarrow p_{t}^{\left(L,i\right)}-1$
18:   $\mathrm{if}\;p_{t}^{\left(L,i\right)}>0$
19:    ${𝓕}_{t}^{i}\leftarrow {\hat{𝓕}}_{t}^{i}$
20:    ${\hat{𝓕}}_{t}^{i}\leftarrow \sqrt{{\left(1-{\hat{F}}_{t}^{\left(H,i\right)}\right)}^{2}+{\left(1-{\hat{F}}_{t}^{\left(L,i\right)}\right)}^{2}}$
21:   **else break**;22:   **end if**23:  **end while**24: **end if**25: **end if**

In Algorithm 1, ${\hat{F}}_{t}^{\left(H,i\right)}$ and ${\hat{F}}_{t}^{\left(L,i\right)}$ are the expected self-fairness values calculated assuming that slots are reallocated. It is assume that $Q^{H}$ gains a slot from $Q^{L}$, hence, ${\hat{F}}_{t}^{\left(H,i\right)}$ is calculated by increasing $p_{t}^{\left(H,i\right)}$ by 1, and ${\hat{F}}_{t}^{\left(L,i\right)}$ is calculated by decreasing $p_{t}^{\left(L,i\right)}$ by 1. The updated $p_{t}^{\left(H,i\right)}$ and $p_{t}^{\left(L,i\right)}$ are transmitted to its 1-hop neighboring nodes at the end of each frame. Accordingly, during slot exchange at frame time t, φ is calculated using only the locally updated $p_{t}^{\left(H,i\right)}$ and $p_{t}^{\left(L,i\right)}$ by intra-node slot exchange. In the next frame, the self-fairness is updated through information exchanges among neighboring nodes. When $p_{t}^{\left(L,i\right)}=1$ and $Q^{L}$ releases a slot, $p_{t}^{\left(L,i\right)}$ will be 0. This makes $\varphi_{t}^{\left(L,i\right)}=0$, and ${\hat{F}}_{t}^{\left(L,i\right)}$ becomes infinity. To prevent this, a minimum default value above 0 is assigned to $p_{t}^{\left(L,i\right)}$ under this situation.

At every frame, slots are reallocated until self-fairness can no longer be improved. Note that the fairness index 1 is the fairest state. Consequently, the Euclidean distance between the fairest status $F_{t}^{\left(H,i\right)}=F_{t}^{\left(L,i\right)}=1$ and a current $\left(F_{t}^{\left(H,i\right)},\;F_{t}^{\left(L,i\right)}\right)$ combination is introduced as a metric representing a target fairness, as it is presented in Equation (4):

(4) $${𝓕}_{t}^{i}=\sqrt{{\left(1-F_{t}^{\left(H,i\right)}\right)}^{2}+{\left(1-F_{t}^{\left(L,i\right)}\right)}^{2}}$$

Now, the expected Euclidean distance ${\hat{𝓕}}_{t}^{i}$ from the expected fairness $\left({\hat{F}}_{t}^{\left(H,i\right)},\;{\hat{F}}_{t}^{\left(L,i\right)}\right)$ is compared with the current Euclidean distance ${𝓕}_{t}^{i}$ from $\left(F_{t}^{\left(H,i\right)},\;F_{t}^{\left(L,i\right)}\right)$. If ${\hat{𝓕}}_{t}^{i}<{𝓕}_{t}^{i}$, $Q^{H}$ gains a slot from $Q^{L}$, and $p_{t}^{\left(H,i\right)}$ and $p_{t}^{\left(L,i\right)}$ are updated. Because slot reallocation is an intra-node process, collisions with 2-hop neighboring nodes need not be considered.

When $F_{t}^{\left(H,i\right)}<F_{t}^{\left(L,i\right)}$, the slot reallocation algorithm is very similar to Algorithm 1, and ${\hat{F}}_{t}^{\left(H,i\right)}$ and ${\hat{F}}_{t}^{\left(L,i\right)}$ are calculated with $p_{t}^{\left(H,i\right)}-1$ and $p_{t}^{\left(L,i\right)}+1$, respectively. However, instead of $p_{t}^{\left(L,i\right)}>0$ in lines 9 and 18 of Algorithm 1, $p_{t}^{\left(H,i\right)}>1$ is used as a slot release condition. This prevents $p_{t}^{\left(H,i\right)}$ from being zero by releasing all slots to $Q^{L}$ to improve the fairness when $q_{t}^{\left(H,i\right)}\ll q_{t}^{\left(L,i\right)}$. That is, $p_{t}^{\left(H,i\right)}\geq 1$ is guaranteed in any situation.

After the intra-node slot reallocation algorithm, the inter-node slots reallocation [25] follows. At this time, the slot exchange does not consider the weights of $Q^{H}$ and $Q^{L}$ any more because these exchanges take place among the queues with the same importance. Node i’s $Q^{A}$ computes $u_{t}^{\left(A,i\right)}$ to determine how many slots to reallocate with a 1-hop neighboring node as it is presented in Equation (5) [25]:

(5) $$u_{t}^{\left(A,i\right)}=\left[q_{t}^{\left(A,k\right)}\cdot \frac{\sum_{k\in N_{i}}p_{t}^{\left(A,k\right)}}{\sum_{k\in N_{i}}q_{t}^{\left(A,k\right)}}\right]-p_{t}^{\left(A,k\right)}$$

If $u_{t}^{\left(A,i\right)}>0$, slots are gained from the 1-hop neighboring node. If $u_{t}^{\left(A,i\right)}<0$, slots are released to the 1-hop neighboring node. The number of reallocated slots is determined by $\min\left\{u_{t}^{\left(A,i\right)},\;u_{t}^{\left(A,i\right)}-u_{t}^{\left(A,k\right)},\;p_{t}^{\left(A,k\right)}\right\}$. This increases the equality of the inverse-load of the same importance among node i and its 1-hop neighboring nodes. These processes are performed for all nodes in a node-by-node manner. The same intra-node and inter-node slot reallocations are repeated in the next frame.

## 4. Performance Evaluation

A network simulator [35] implemented in Java was used for performance analysis of the proposed algorithm. No isolated nodes are assumed, i.e., all the nodes have at least a single 1-hop neighbor node. Accordingly, in establishing a connection, any two nodes can be connected with each other through multi-hop links. The connections are established using arbitrarily chosen pairs of a source node and a destination node, and high- and low-importance connections generate high- and low-importance packets, respectively. In the following, high- and low-importance packets are denoted by ${\mathrm{Pkt}}^{H}$ and ${\mathrm{Pkt}}^{L}$, respectively.

For the performance analysis, the throughput, delay, and fairness are measured by changing the connection creation ratio (between ${\mathrm{Pkt}}^{H}$ and ${\mathrm{Pkt}}^{L}$) and the weight factor setting. Then, the proposed algorithm is compared with the absolute priority-based algorithm in which ${\mathrm{Pkt}}^{H}$ preempts time slots when allocating free time slots. Note that the absolute priority algorithm adopts only the inter-node slot reallocation algorithm, not the intra-node slot one.

The generation ratios of high- and low-importance connections are denoted by α, 1−α∈[0,1]. The weight factor setting in $Q^{A}$ is denoted by $r^{A}$. Assuming that $Q^{H}$ and $Q^{L}$ of all nodes have the same weight settings as $r^{H}$ and $r^{L}$, respectively, the node index i can be dropped from the weight factors. The weight factors are set as: $r^{H},\;r^{L}\in \left[0,10\right]$ and $r^{H}+r^{L}=10$.

The performance of the proposed scheme was measured in two scenarios. Table 2 lists the parameters setting for each scenario. In the first scenario, a fixed number of connections are created at the starting epoch of the simulation, the packets of the connections are generated at fixed time intervals, and the number of packets generated for each connection is the same. In the second scenario, connections are created based on Poisson processes. Unlike the first scenario, the number of packets generated per connection follows a Poisson distribution. The arrival rate λ determines the connection creation interval. The duration of each connection follows an exponential distribution of parameter μ, which determines the number of packets generated in each connection. The packets are generated at a fixed interval, as in the first scenario. Each connection is closed if all the packets arrive at its destination node. Because the connections are continually generated, in the second scenario, the simulation duration is specified at the beginning of the simulation. For both scenarios, the final measurement is the average over 1000 independent simulations.

In the first scenario, the performance of the proposed algorithm was analyzed with the increasing total number of connections and the various settings of the weight factor and α. The total number of created connections is the sum of the high- and low-importance connections. Throughput, packet delivery ratio, 1-hop delay, and fairness are measured and compared with those of absolute priority-based scheduling. Throughput refers to the number of all packets arriving at a destination node during the simulation. However, in the first scenario, since the number of generated connections is determined at the beginning of the simulation, the throughput measured when all packets arrive at a destination node will be simply the product of $N_{c}$ (number of connections) and $N_{p}$ (number of generated packets per connection). Therefore, throughput is measured not at the end of the simulation but at a predefined time T, which is large enough for the transmission of packets in the network to be in a steady state. The packet delivery ratio means the proportion of received packets to the packets sent. The 1-hop delay is measured as the average of ((the time when a packet is dequeued) minus (the time when a packet is enqueued)). The results of the absolute priority-based algorithm are marked as $\mathrm{Preempt}.{\mathrm{Pkt}}^{H}$ and $\mathrm{Preempt}.{\mathrm{Pkt}}^{L}$.

**Figure 2:**
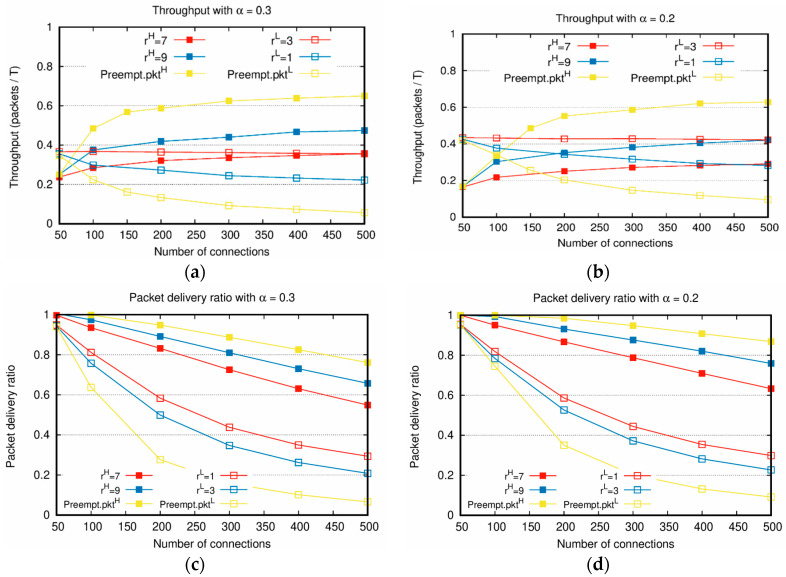
Throughput comparisons between ${\mathrm{Pkt}}^{H}$ and ${\mathrm{Pkt}}^{L}$ with increasing number of connections: (**a**,**b**) throughputs with α=0.3, α=0.2; (**c**,**d**) packet delivery ratios with α=0.3, α=0.2.

**Figure 3:**
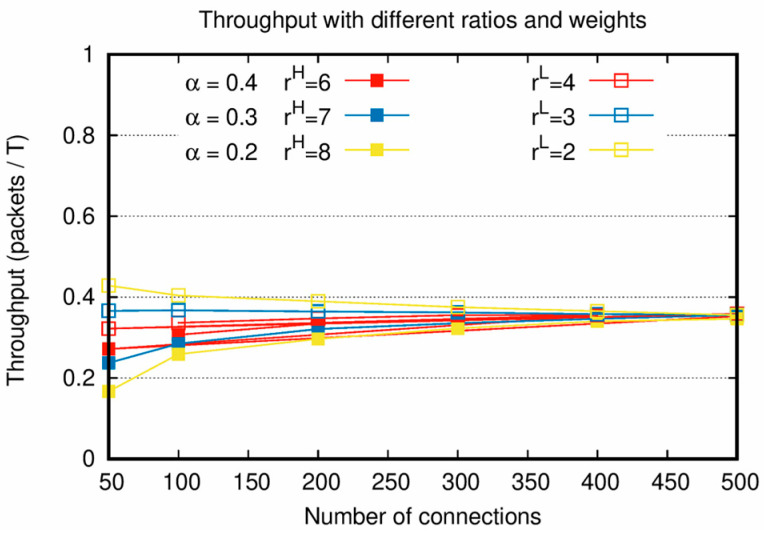
Throughput with various connection creation ratios and weight factors with increasing number of connections.

**Figure 4:**
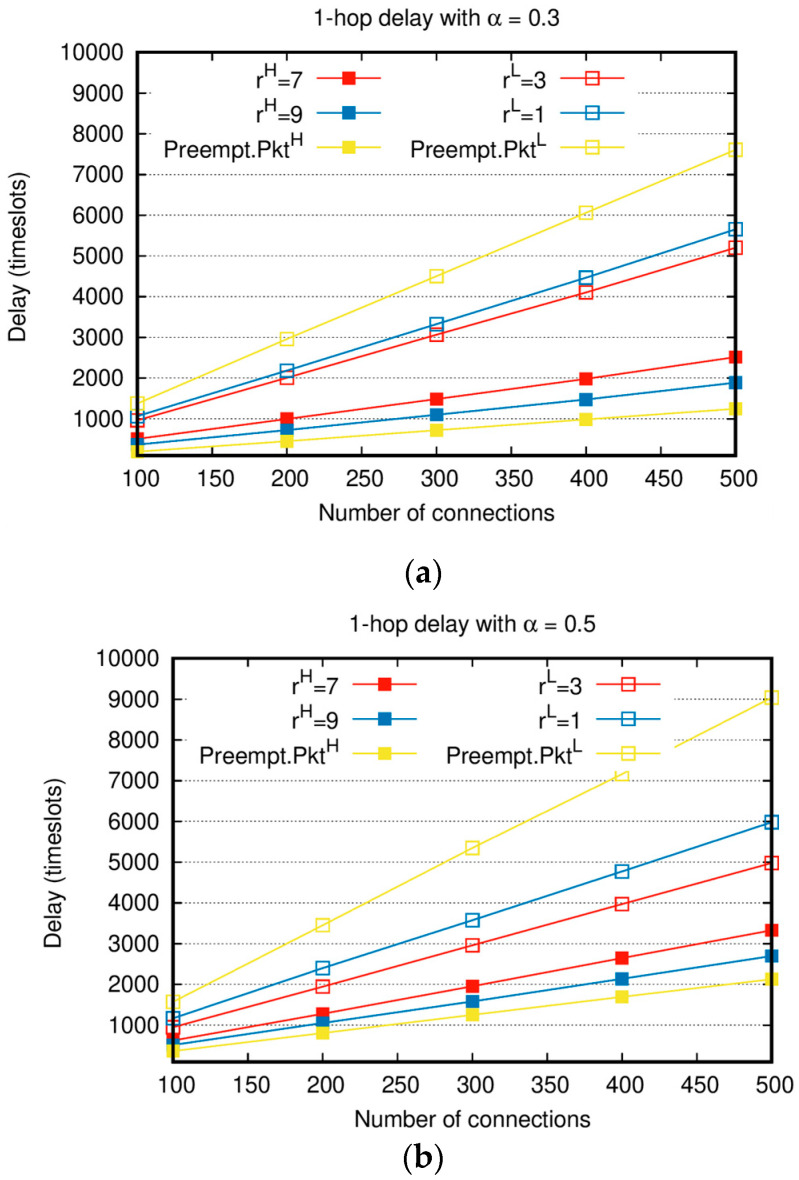
Delay comparison between priority-based algorithms ${\mathrm{Pkt}}^{H}$ and ${\mathrm{Pkt}}^{L}$: (**a**) 1-hop delay with α=0.3; (**b**) 1-hop delay with α=0.5.

**Figure 5:**
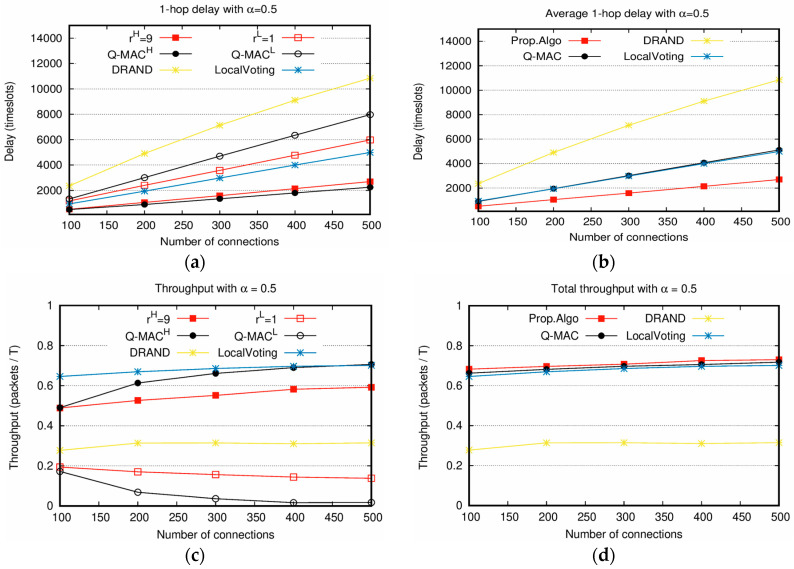
Delay and throughput comparison between the proposed algorithm and other scheduling algorithms with α=0.5: (**a**,**b**) 1-hop delays of different weight values and average 1-hop delay; (**c**,**d**) throughputs with different weight values and total throughputs.

**Figure 6:**
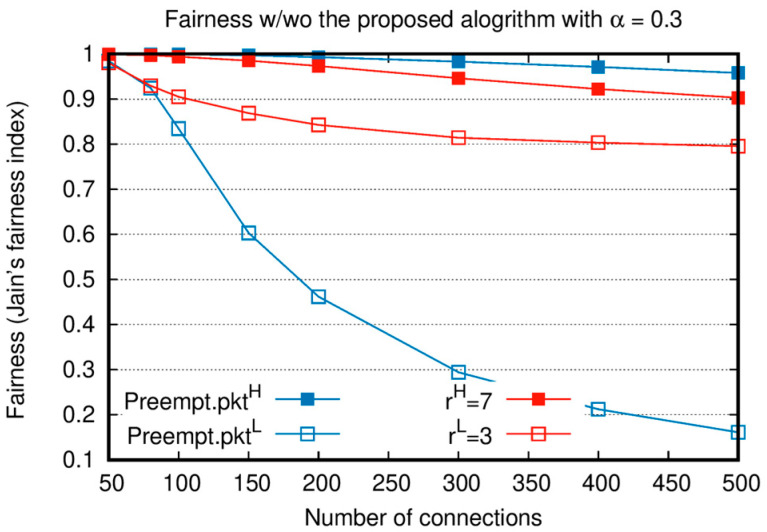
Jain’s fairness comparison between the proposed algorithm and absolute priority-based scheduling.

Figure 2, Figure 3, Figure 4, Figure 5 and Figure 6 show the results of the first scenario. Figure 2 depicts the throughputs with the increasing total number of connections, various weight factors, and α=0.3. When the number of connections is small, most packets are delivered to the destination nodes until the predefined time T because the network is not heavily loaded. For this reason, in Figure 2a,b, when the number of connections is 50, the throughput of ${\mathrm{Pkt}}^{H}$ is lower than that of ${\mathrm{Pkt}}^{L}$ because the number of ${\mathrm{Pkt}}^{H}$ is lower than ${\mathrm{Pkt}}^{L}$. In most cases, if the number of connections increases, the throughput of ${\mathrm{Pkt}}^{H}$ is higher than that of ${\mathrm{Pkt}}^{L}$. However, in Figure 2b, when the weight factors are $r^{H}=7$ and $r^{L}=3$, the throughput of ${\mathrm{Pkt}}^{L}$ is higher than that of ${\mathrm{Pkt}}^{H}$, even when the number of connections increases. Note that the proposed algorithm considers not only the weight factors but the traffic load as well; hence, even when $r^{L}<r^{H},$ the throughput of ${\mathrm{Pkt}}^{L}$ is higher than that of ${\mathrm{Pkt}}^{H}$ in the entire range of $N_{c}$. The service differentiation between ${\mathrm{Pkt}}^{H}$ and ${\mathrm{Pkt}}^{L}$ is more evidently shown in Figure 2c,d. As shown in these figures, over all the range of the number of connections, the packet delivery ratio of ${\mathrm{Pkt}}^{H}$ is higher than ${\mathrm{Pkt}}^{L}$. Specifically, Figure 2b with $r^{H}=7$, $r^{L}=3$ can be compared with Figure 2d with $r^{H}=7$, $r^{L}=3$. In this case, Figure 2b shows that the throughput of ${\mathrm{Pkt}}^{L}$ is higher than ${\mathrm{Pkt}}^{H}$. However, Figure 2d shows that the packet delivery ratio of ${\mathrm{Pkt}}^{H}$ is still twice as high as that of ${\mathrm{Pkt}}^{L}$. This result clearly shows that the proposed scheme preferentially processes packets reflecting the weight factors. When the absolute priority-based algorithm is applied, as the number of ${\mathrm{Pkt}}^{H}$ to be transmitted increases owing to the increment of the number of connections, the opportunity for ${\mathrm{Pkt}}^{L}$ slot allocation decreases, resulting in a further decrease in the throughput of ${\mathrm{Pkt}}^{L}$.

In Figure 3, throughputs are measured when $r^{H}\cdot \alpha =r^{L}\cdot \left(1-\alpha \right)$ is satisfied under the condition of increasing number of connections. Figure 3 shows the characteristics of the proposed algorithm by considering both the weight factor and traffic load. When $r^{H}\cdot \alpha =r^{L}\cdot \left(1-\alpha \right)$ is satisfied, it is confirmed that the throughputs of ${\mathrm{Pkt}}^{H}$ and ${\mathrm{Pkt}}^{L}$ have similar values and converge to a single value, as shown in Figure 3.

As shown in Figure 2 and Figure 3, the sums of the throughputs of ${\mathrm{Pkt}}^{H}$ and ${\mathrm{Pkt}}^{L}$ are similar when $N_{c}$ is the same, even though α and the weight factors are different. This is because even when the number of allocated slots of ${\mathrm{Pkt}}^{H}$ and ${\mathrm{Pkt}}^{L}$ are changed by α and the weight factors during the process of reallocation, the number of allocated slots in the entire network does not change. Therefore, there is a tradeoff between the throughputs of ${\mathrm{Pkt}}^{H}$ and ${\mathrm{Pkt}}^{L}$ depending on the weight factors. From Figure 2 and Figure 3, it is confirmed that an appropriate weight factor setting is necessary to adjust the throughputs of ${\mathrm{Pkt}}^{H}$ and ${\mathrm{Pkt}}^{L}$ for various network situations with different α.

Figure 4 shows 1-hop delay with various weight factors and α with the increasing total number of connections. Similar to in Figure 2 and Figure 3, when the number of connections is small, all the generated packets can be delivered to destination nodes, resulting in nearly no difference in the delay between ${\mathrm{Pkt}}^{H}$ and ${\mathrm{Pkt}}^{L}$. However, as the number of connections increases, the delays of both ${\mathrm{Pkt}}^{H}$ and ${\mathrm{Pkt}}^{L}$ increase, and the delay difference between ${\mathrm{Pkt}}^{H}$ and ${\mathrm{Pkt}}^{L}$ becomes conspicuous. Compared to the absolute priority-based algorithm, the delay gap between ${\mathrm{Pkt}}^{H}$ and ${\mathrm{Pkt}}^{L}$ of the proposed algorithm is relatively small. In the case of $r^{H}=7$ and $r^{L}=3$ shown in Figure 4a, when $N_{c}$ is 500, the delay of ${\mathrm{Pkt}}^{L}$ is twice that of ${\mathrm{Pkt}}^{H}$. On the other hand, the delay of Preempt. ${\mathrm{Pkt}}^{L}$ is more than 6 times the delay of $\mathrm{Preempt}.{\mathrm{Pkt}}^{H}$. The delay of ${\mathrm{Pkt}}^{H}$ increases compared to Preempt.${\mathrm{Pkt}}^{H}$, but the delay of ${\mathrm{Pkt}}^{L}$ decreases much more than Preempt.${\mathrm{Pkt}}^{L}$. In particular, when $r^{H}=9,r^{L}=1$, and $N_{c}=500$ in Figure 4b, the delay of ${\mathrm{Pkt}}^{H}$ increases by approximately 500 time slots compared to $\mathrm{Preempt}.{\mathrm{Pkt}}^{H}$, but the delay of ${\mathrm{Pkt}}^{L}$ decreases by approximately 3000 time slots compared to $\mathrm{Preempt}.{\mathrm{Pkt}}^{L}$, and it is a noticeable improvement. The average sum delay of ${\mathrm{Pkt}}^{H}$ and ${\mathrm{Pkt}}^{L}$ is reduced by 20% compared to that of $\mathrm{Preempt}.{\mathrm{Pkt}}^{H}$ and $\mathrm{Preempt}.{\mathrm{Pkt}}^{L}$. This means that, compared to the absolute priority-based algorithm, the proposed algorithm achieves the higher performance. Moreover, the proposed algorithm can achieve the same delay performance with $\mathrm{Preempt}.{\mathrm{Pkt}}^{H}$ by throttling ${\mathrm{Pkt}}^{L}$, i.e., with $r^{H}=10$ and $r^{L}=0$. When α=0.5, the number of ${\mathrm{Pkt}}^{H}$ to be transmitted increases and the delay of ${\mathrm{Pkt}}^{H}$, at the same $N_{c}$, increases compared to the case of α=0.3. In the whole range of $N_{c}$, the delay of ${\mathrm{Pkt}}^{H}$ in Figure 4b is higher than that of ${\mathrm{Pkt}}^{H}$ in Figure 4a. In addition, ${\mathrm{Pkt}}^{H}$‘s delay when $r^{H}=7$ in Figure 4a and that when $r^{H}=9$ in Figure 4b are similar.

In Figure 2 and Figure 4, for ${\mathrm{Pkt}}^{H}$, the higher $r^{H}$ is, the better the performances of throughput and delay are. The decrement in $r^{L}$ due to the increased $r^{H}$ leads to the worse performance of throughput and delay of ${\mathrm{Pkt}}^{L}$. The larger the difference between the values of $r^{H}$ and $r^{L}$, the larger the performance gap between the throughput and delay of ${\mathrm{Pkt}}^{H}$ and ${\mathrm{Pkt}}^{L}$. This confirms that ${\mathrm{Pkt}}^{H}$ and ${\mathrm{Pkt}}^{L}$ are flexibly adjusted based on the values of the weight factor in various network situations.

In Figure 5, the proposed scheduling scheme is compared with DRAND, LocalVoting, and Q-MAC. Q-MAC was developed for CSMA/CA and the packets with high weight value had a relatively high probability of accessing channel. For comparison, Q-MAC was modified to be applicable to TDMA. Specifically, the slots of Q-MAC are initialized according to the weight values, and the inter-node reallocation of LocalVoting is followed. As shown in Figure 5a, the delay of ${\mathrm{Pkt}}^{H}$ is better than both DRAND and LocalVoting, and slightly worse than Q-MAC with ${\mathrm{Pkt}}^{H}$. Even ${\mathrm{Pkt}}^{L}$ shows the better performance than DRAND and slightly worse than LocalVoting. Specifically, the delay of DRAND is twice longer than ${\mathrm{Pkt}}^{L}$ and four times longer than ${\mathrm{Pkt}}^{H}$. LocalVoting shows the performance better than DRAND through the neighbor-aware load balancing. However, the proposed scheme of ${\mathrm{Pkt}}^{H}$ still outperforms LocalVoting. The delay of ${\mathrm{Pkt}}^{H}$ is 1.8 times smaller than LocalVoting. In Figure 5b, the average delay of the proposed scheme shows the best performance. Q-MAC and LocalVoting show the similar performance with each other. In Figure 5c, the throughput of the proposed scheme with ${\mathrm{Pkt}}^{H}$ lower than Q-MAC with ${\mathrm{Pkt}}^{H}$. However, the throughput of the proposed scheme with ${\mathrm{Pkt}}^{L}$ is higher than Q-MAC with ${\mathrm{Pkt}}^{L}$. Note that the throughput of LocalVoting in Figure 5c is the sum of its ${\mathrm{Pkt}}^{H}$ and ${\mathrm{Pkt}}^{L}$. In Figure 5d, the proposed scheme achieves the highest throughput. In Figure 5b,d, it is ensured that the proposed scheme possesses the excellent performance in slot allocation because it achieves the highest throughput and the lowest delay. 

Moreover, Figure 5a,c show that the service differentiation of the proposed scheme is enabled compared with other schemes. These are the major contributions of the proposed scheme.

Figure 6 compares Jain’s fairness [20] of ${\mathrm{Pkt}}^{H}$ and ${\mathrm{Pkt}}^{L}$ with and without the proposed algorithm. In this figure, in terms of $\Gamma^{\left(A,i\right)}$, Jain’s fairness index shows how fairly resources are allocated among the queues of the same importance. $\Gamma^{\left(A,i\right)}$ is the ratio of the accumulative number of packets transmitted from a queue to the number of accumulated packets in a queue until T, which can be expressed as Equation (6). Similar to the throughput measurement, at the end of the simulation, all packet delivery is completed; accordingly, Jain’s fairness is calculated at time T.

(6) $$\Gamma^{\left(A,i\right)}=\frac{\sum_{t=0}^{T}p_{t}^{\left(A,i\right)}}{\sum_{t=0}^{T}q_{t}^{\left(A,i\right)}},\;A\in \left\{H,L\right\}$$

In this analysis, α=0.3 and $r^{H}=7$, $r^{L}=3$ are considered. When the number of connections is small, the fairness index is high regardless of the adoption of the proposed algorithm because the $\Gamma^{\left(A,i\right)}$ of most nodes becomes close to 1. For the absolute priority-based algorithm, as the number of connections increases, only a few nodes are allocated slots for Preempt.${\mathrm{Pkt}}^{L}$. Since most nodes cannot transmit Preempt.${\mathrm{Pkt}}^{L}$, the fairness of Preempt.${\mathrm{Pkt}}^{L}$ is very low. In contrast, when the intra-node slot reallocation of the proposed algorithm is adopted, time slots proportional to $r^{L}$ are allocated to $Q^{L}$, and this results in an increase in the fairness index. As a result, the fairness performance of ${\mathrm{Pkt}}^{L}$ is significantly increased compared to that of ${\mathrm{Pkt}}^{H}$ when the intra-node slot exchange algorithm is applied.

**Figure 7:**
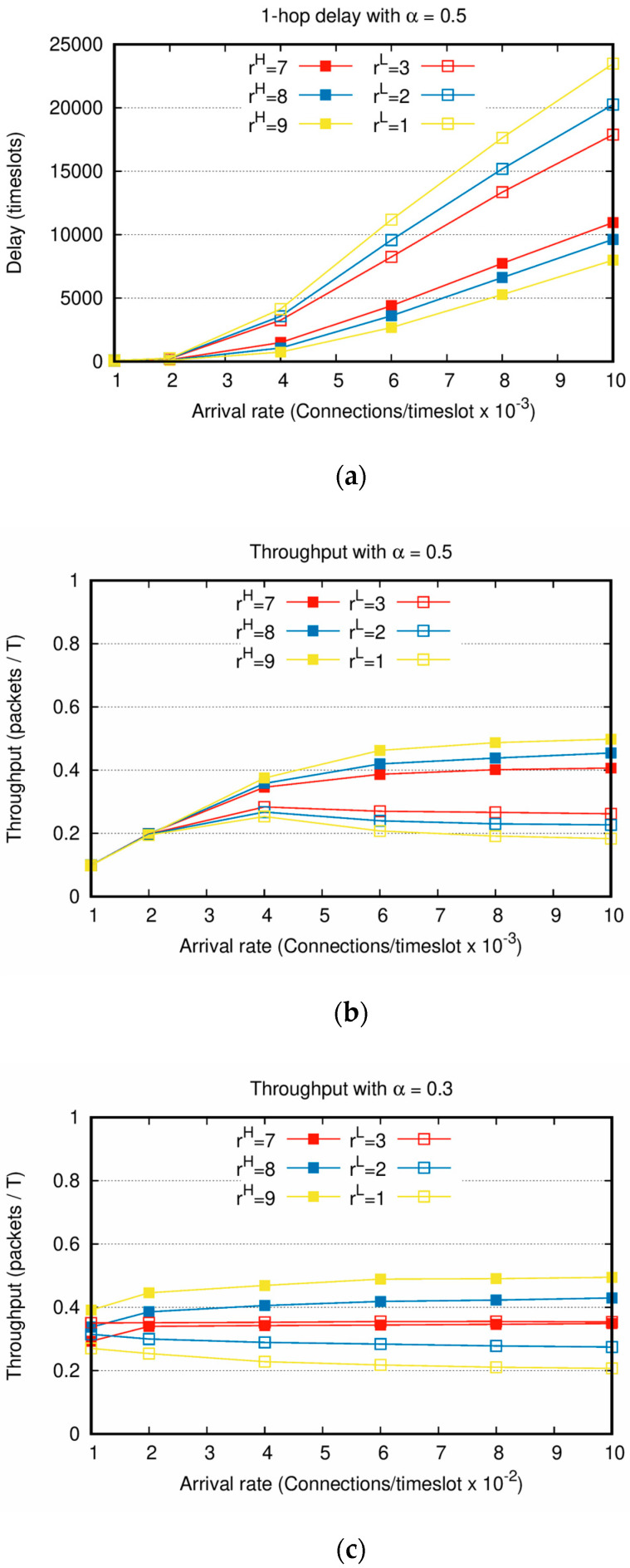
Delay and throughput with increasing Poisson arrival rates and the same weight factor setting: (**a**) 1-hop delay with α=0.5; (**b**) throughput with α=0.5; (**c**) throughput with α=0.3.

Figure 7 shows the delay and throughput performance of the second scenario with the increasing Poisson arrival rate λ. In Figure 7a,b, because α=0.5 is applied, the numbers of ${\mathrm{Pkt}}^{H}$ and ${\mathrm{Pkt}}^{L}$ are similar. Although the connection creation interval and the number of packets generated for each connection are varied, Figure 7 shows similar performances to those of the first scenario. The larger the difference between $r^{H}$ and $r^{L}$, the greater the performance gap between ${\mathrm{Pkt}}^{H}$ and ${\mathrm{Pkt}}^{L}$. For instance, in Figure 7a, when the arrival rate is 0.01 $\mathrm{time}-{\mathrm{units}}^{-1}$ and the weight factors are $r^{H}=7$ and $r^{L}=3$, the ${\mathrm{Pkt}}^{L}$ delay is approximately 1.5 times longer than the ${\mathrm{Pkt}}^{H}$ delay. However, when the weight factors are $r^{H}=9$ and $r^{L}=1$, ${\mathrm{Pkt}}^{L}$ delay is over two times ${\mathrm{Pkt}}^{H}$ delay. When the arrival rate is low, the connection creation interval is long, and the number of connections created during the entire simulation is small. As shown in Figure 7a,b, when the arrival rates are as low as 0.001 and 0.002 $\mathrm{time}-{\mathrm{units}}^{-1}$, there is only a slight difference in delay and throughput between ${\mathrm{Pkt}}^{H}$ and ${\mathrm{Pkt}}^{L}$ regardless of the weight factor setting.

Figure 7c shows the throughput when the number of ${\mathrm{Pkt}}^{L}$ is larger than that of ${\mathrm{Pkt}}^{H}$, by setting α=0.3. The result of Figure 7c is very similar to that of Figure 2a when $N_{c}$ ranges between 100 and 500. In particular, if $r^{H}\cdot \alpha =r^{L}\cdot \left(1-\alpha \right)$ is satisfied by setting $r^{H}=7,\;r^{L}=3$, the throughputs of ${\mathrm{Pkt}}^{H}$ and ${\mathrm{Pkt}}^{L}$ converge to a constant value. However, note that α is set as 0.3, i.e., 70% of the generated packets are ${\mathrm{Pkt}}^{L}$ and the remaining 30% is ${\mathrm{Pkt}}^{H}$. Even in this asymmetric packet generation scenario, ${\mathrm{Pkt}}^{H}$ achieves the higher throughput than ${\mathrm{Pkt}}^{L}$. Accordingly, this clearly shows that the service differentiation between ${\mathrm{Pkt}}^{H}$ and ${\mathrm{Pkt}}^{L}$ is attained.

## 5. Conclusions

In this paper, a novel distributed node scheduling algorithm for an ad-hoc network was proposed. This scheme flexibly adjusts time slot allocations according to weight factor and traffic load. From thorough simulation studies under various environments, the performance differentiation reflecting weight factor setting was validated. It was confirmed that, as the weight of the high importance packets increases, the delay decreases and the throughput at the same time increases. Because the proposed algorithm considers both the weight factors and traffic loads, even the throughput and delay for the same weight factors can be adjusted separately according to the connection creation ratios with different importance. Through comparison with other distributed node scheduling algorithms, the advantages of the proposed algorithm were validated. Specifically, it supports load balancing with neighboring nodes and preferential processing of important data. In addition, compared to the conventional absolute priority-based algorithm, the proposed algorithm shows performance improvement in terms of throughput, delay, and fairness for low-importance packets. Moreover, the performance comparison with other scheduling scheme ensures the excellent performance of the proposed scheme because it achieves the highest throughput and the lowest delay. These results verify that both the service differentiation and performance improvement can be achieved through an appropriate weight factor setting.

## Figures and Tables

**Table 1:** Comparative analysis of related works.

Classification	Protocol	Access Mechanism	Load-Balancing	Weight Factor	Goal
Without Prioritization	DRAND [22]	TDMA	No	N/A	To allocate resources efficiently in ad-hoc networks
	LocalVoting [25]	TDMA	High	N/A	To decrease average delay by making the load between neighbor nodes semi-equal
	ATLAS [26]	TDMA	High	N/A	To adapt topology changes fast and allocate resources considering neighbor nodes
With Prioritization	ELBPQA [29]	CSMA/CA	Low	No	To minimize delay of high priority packets
	Algo. [30]	TDMA	Low	No	To minimize end-to-end delay of high priority packets
	I-MAC [32]	CSMA + TDMA	Medium	No	To increase chance of resource allocation for high priority nodes by CSMA + TDMA
	PAQMAC [31]	CSMA/CA	No	Quantitative	To allocate active time dynamically by considering the priority of packets
	Q-MAC [33]	CSMA/CA	Medium	Quantitative	To increase energy efficiency while providing service differentiation

**Table 2:** Simulation parameters.

Parameter	Value
Number of nodes	30
Transmission range	5 units
Topology size	50 × 50 units
Frame length	30 time-units
Packet generation interval	5 time-units
Number of connections $N_{c}$	50–500
Number of packets per connection $N_{p}$	50
Connection duration μ	$10^{3}$ time-units
Arrival rate λ	$10^{-3}–10^{-1}$ time-${\mathrm{units}}^{-1}$
Simulation time T (first/second scenario)	3000/150,000 time-units
